# Importance of Build Design Parameters to the Fatigue Strength of Ti6Al4V in Electron Beam Melting Additive Manufacturing

**DOI:** 10.3390/ma15165617

**Published:** 2022-08-16

**Authors:** Sean Ghods, Reid Schur, Alex Montelione, Rick Schleusener, Dwayne D. Arola, Mamidala Ramulu

**Affiliations:** 1 Department of Materials Science and Engineering, University of Washington, Seattle, WA 98195, USA; 2 Department of Mechanical Engineering, University of Washington, Seattle, WA 98195, USA

**Keywords:** additive manufacturing, defects, electron beam melting, fatigue, porosity, titanium, X-ray computed microtomography

## Abstract

The fatigue properties of metals resulting from Powder Bed Fusion (PBF) is critically important for safety-critical applications. Here, the fatigue life of Grade 5 Ti6Al4V from Electron Beam PBF was investigated with respect to several build and component design parameters using a design of experiments (DOE). Part size (i.e., diameter), part proximity, and part location within the build envelope were considered. Overall, metal in the as-built condition (i.e., no post-process machining) exhibited a significantly lower fatigue life than the machined surface condition. In both conditions, the fatigue life decreased significantly with the decreasing part diameter and increasing radial distance; height was not a significant effect in the machined condition. Whereas the surface topography served as the origin of failure for the as-built condition, the internal lack of fusion (LOF) defects, exposed surface LOF defects, and rogue defects served as the origins for the machined condition. Porosity parameters including size, location, and morphology were determined by X-ray micro-computed tomography (XCT) and introduced within regression models for fatigue life prediction. The greatest resistance to fatigue failure is obtained when parts are placed near the center of the build plane to minimize the detrimental porosity. Machining can improve the fatigue life, but only if performed to a depth that minimizes the underlying porosity.

## 1. Introduction

The titanium alloy Ti6Al4V is commonly employed in the aerospace industry for stress-critical applications, particularly those involving cyclic loading. Wrought forming processes followed by selected secondary processes have been the standard for achieving an exceptional fatigue performance for this alloy. With the progressive advancement of metal additive manufacturing (AM) in aerospace through powder bed fusion (PBF), the fatigue performance of the AM parts of Ti6Al4V has become a hot topic. The importance of fatigue and potential contribution of intrinsic defects resulting from the AM processing of Ti6Al4V has motivated research on this subject. In fact, reviews of the high cycle fatigue (HCF) life for Laser and Electron Beam PBF processes are becoming available [1,2,3,4,5,6].

There are specific factors of concern in PBF that could affect the fatigue performance of components, including their location and orientation in the build, as well as the surface finish [1]. The comparatively rough surfaces associated with PBF are the first concern, particularly for Electron Beam Melting (EBM) AM. Sharp notches posed by the surface texture due to the unmelted and irregularly spread powder leads to premature fatigue crack initiation at the surface [7,8,9]. While post-process machining to eliminate the notch effects could significantly improve the cycles to failure, it is undesirable for many reasons.

Component orientation within the build is the second major factor of relevance to the fatigue performance. The orientation dependence is related to the microstructure, defects, and their distribution [1]. Internal defects are detrimental due to the inherent stress concentration and are more problematic in parts printed with a vertical orientation (along the build direction) relative to the horizontal orientation [1,3]. The major axis of the lack of fusion (LOF) voids in metal AM parts is typically within the build plane, which results in the largest dimension being oriented perpendicular to the maximum normal stress. The largest pores (in the metal volume) tend to be a result of incomplete fusion between layers [10]. As such, the stress concentration posed by these pores can be of greater importance than other microstructural features and leads to the discrepancy between vertically and horizontally printed parts [3]. Internal defects are a significant concern, which has motivated research and the development of predictive models capable of relating the defect characteristics to the fatigue properties [11,12,13,14]. While the physical size of these voids is commonly considered to be the dominant attribute [15], there are other characteristics that should be considered, including the shape [16], location relative to the surface [11,17], and location relative to other pores/defects [7]. Each of these characteristics can contribute to the stress concentration posed by an internal pore.

Key to developing high quality metal with AM is understanding the property variability and which parameters that potentially compromise the performance. Apart from the process parameters, the build design parameters are of substantial importance, but are often ignored. For instance, part placement within the build space and its geometry were found to have significant effects on the mechanical properties under quasi-static tension [18]. Similarly, these build design parameters are expected to have comparable effects on the high cycle fatigue (HCF) response. However, studies concerning the effects of build design parameters on the fatigue performance of Ti6Al4V or other metals are scant. In a study concerning the fatigue response of Ti6Al4V and the contribution of specimen geometry, the fatigue life increased with component thickness [19]. For thicker specimens, the surface defects were less detrimental, which reduced the incidence of crack initiation from the surface. Furthermore, the alpha lath thickness increased with part thickness, which also increased the crack growth resistance [2]. A related study reported a similar trend with the geometry and fatigue life for the “as-built condition”, which was attributed to a higher surface roughness exhibited by components with a lower thickness [8]. Nevertheless, these studies did not consider the effects of geometry in the machined state, or after post-processing surface treatment, which will typically be adopted for components intended for stress-critical applications. The present study evaluates the contribution of build design effects, including part geometry and its location, to the fatigue performance of the Ti6Al4V components produced by EBM. This is a novel contribution with substantial industrial relevance.

## 2. Materials and Methods

### 2.1. Powder Feedstock

The powder used in this study consisted of Grade 5 Titanium alloy (Ti6Al4V), acquired from the EBM manufacturer (ARCAM (Mölndal, Sweden): Batch P1143, Part #430944). The original powder lot consisted of 100 kg of virgin powder and was used in two builds, as reported in Ghods et al., [18] prior to the present investigation; the remaining lot of powder was approximately 95 kg. Here, three builds were performed in total to evaluate the metal fatigue properties in the “as-built” (tested as built without post-process machining) and “machined” conditions. Although it is customary to mix virgin powder in between each build to maintain powder volume and quality, no additional powder was added to the lot during this study. Briefly, after completion of each build and prior to the present investigation, loose powder was removed from the build chamber using a dedicated vacuum and added to the volume of powder remaining in the hoppers. The partially sintered powder was removed from the build using the Powder Recovery System (PRS), and then added to the unsintered powder from the chamber and hoppers. This powder volume was manually mixed for approximately 15 min to ensure that thorough mixing was achieved. Thereafter, the mixed powder was passed through a sieve with a 120 mesh size, to remove any large particles (>125 µm) and achieve further mixing. This powder was transferred back into the hoppers in the build chamber for the remaining build.

### 2.2. Build Design

**Figure 1:**
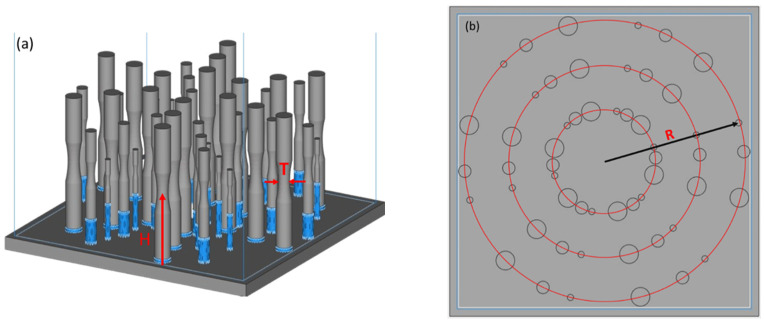
Build design for the preliminary investigation. (**a**) Oblique view of the build design for the as-built fatigue study. Only one height (H) level was printed for this preliminary investigation. Thickness (T) of the parts is the diameter of the thinnest part of the gauge section. (**b**) Top view of the build with red rings indicating the three levels of radial distance (R).

A multi-factor experimental approach was adopted to investigate the effects of selected design parameters and their interactions on the microstructure and fatigue properties of Ti6Al4V. Three factors were investigated including part height in the build volume, radial distance from the center of the build plate, and part thickness (equivalent to diameter), as represented in Figure 1.

Regarding the build design parameters, height refers to the location of the thinnest cross-section of the gauge section along the build direction and radial distance is the location of the central axis of the specimen relative to the center of the build space in the X–Y plane (build plane). Radial distance was selected, as opposed to X and Y position, to reduce the dimensionality of the parameter space investigated. A reduction of the number of factors improves the statistical power of the data. Finally, the component thickness was also considered, which was reflected by the diameter of the thinnest area of the gauge section.

Factor levels used for the DOE of metal evaluated in the “as-built” condition are listed in Table 1. A preliminary study was necessary to develop an understanding of the range in fatigue life responses that resulted from the parameter space explored. The height factor was chosen to be limited to a single level in this preliminary study since it was the least influential factor of the three that were evaluated in the study involving monotonic loading. The other two design factors have three levels to allow for polynomial regression and potential optimization of the parameters.

**Figure 2:**
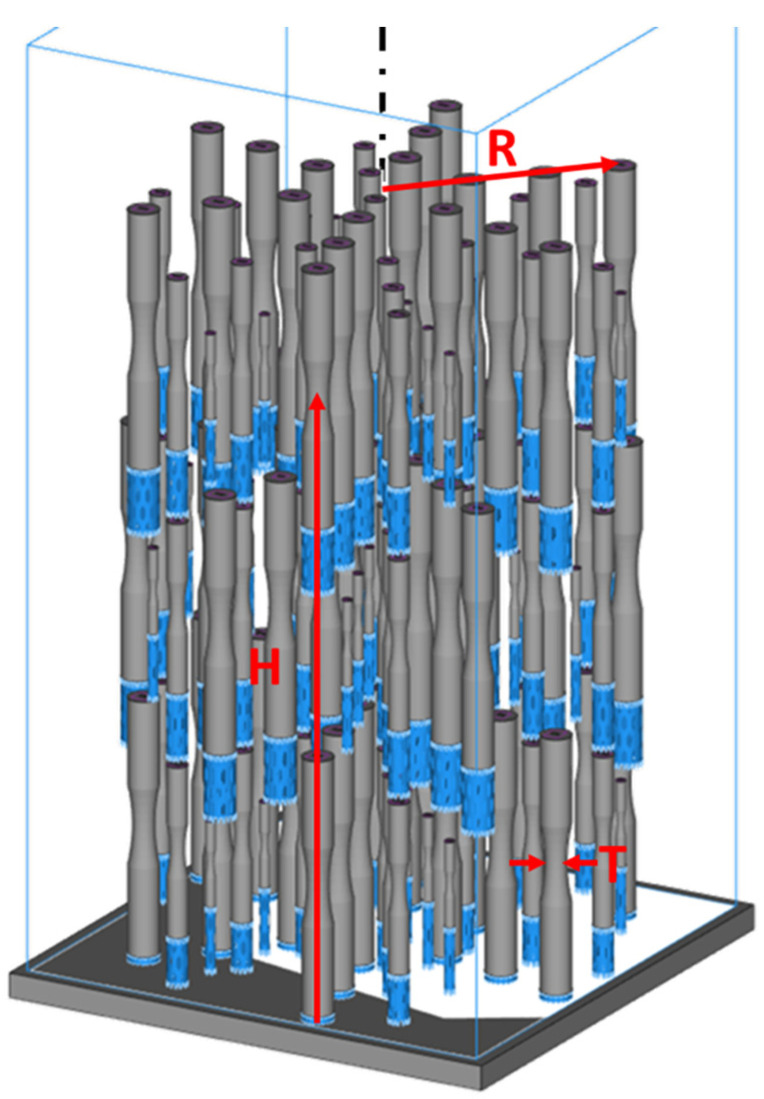
Oblique view of the build for fatigue specimens of the full factorial DOE. Illustrated with the red arrows are the three design parameters—Height (H): distance from the base plate to centroid of a part; Radial distance (R): distance from the middle of the build plane (central axis represented by dashed black line) to centroid of a part; and Thickness (T): diameter of the thinnest portion of the gauge section of a part.

The height, radial distance, and thickness were selected for the preliminary DOE and a second DOE that included an evaluation of the fatigue performance of the metal after post-build machining. The build design adopted for this second DOE is shown in Figure 2. In contrast to the preliminary DOE involving fatigue performance in the as-built condition, a full complement of part height was included across the build, which resulted in a full factorial DOE with 3 factors. Full factorial designs with 3 levels allowed for every combination of factors to be investigated with polynomial regression fits. The non-linear responses reflected in the curve fit can help to identify optimal settings for the selected parameters.

Part proximity, also regarded as part packing density, was not included as a factor of importance in the DOE. Results from prior work involving monotonic loading showed that it had no effect on the mechanical properties [18].

### 2.3. Printing and Design Restrictions

The printing process was conducted with an ARCAM A2X Electron Beam AM system (Mölndal, Sweden). Printing was conducted according to the default parameters of the machine for Ti6Al4V, which includes beam speed of 4530 mm/s, beam current of 15 mA, max current of 20 mA, focus offset of 25 mA, and speed function of 45. The builds were performed with version 5.0.64 of the ARCAM EBM (Mölndal, Sweden) control software and with the machine operated in manual mode (i.e., static parameters).

Part orientation is an important factor to consider in metal AM due to the anisotropic nature of the microstructure in terms of grain orientation and void orientation, as previously described. Despite this concern, only the vertical orientation (along the build direction) was considered in the evaluation of fatigue properties. This orientation was the only feasible option from a buildability perspective, and, more importantly, the vertical orientation is expected to exhibit inferior fatigue life relative to the horizontal orientation [1,20]. The primary orientation of defects (both surface and subsurface) in EBM are within the build plane, which will be the limiting factor for fatigue life of the vertical orientation. Furthermore, packing specimens within the desired locations in the build envelope according to the DOE precludes them from being oriented off axis from the build direction. Furthermore, most components will experience multiaxial stress states with at least a portion of the volume having stresses aligned with the vertical orientation, i.e., perpendicular to the most prominent defects in EBM. Thus, the vertical orientation is the best choice for considering the fatigue performance of metal from EBM.

For the DOE performed with metal in the machined condition, it was not possible to produce enough specimens in a single build to have sufficient sample population as required for a full factorial at the four stress levels of fatigue testing. Therefore, a second build was performed with the exact same build design shown in Figure 2 to mitigate inter-build variation. This second build was conducted immediately following the first build to minimize potential effects of machine condition and/or powder degradation.

### 2.4. Post Processing

The specimens for the full factorial DOE were evaluated in the “machined condition”. Machining was performed to remove effects from the surface roughness on the fatigue life. All parts in the machined condition were processed by a qualified source (Limited Productions Inc., Bellevue, WA, USA) via turning operations with appropriate settings for titanium to avoid microstructural changes or residual stresses at the surface. Approximately 0.35 mm was removed from the external diameter from the entire length of the samples. This resulted in a 0.7 mm decrease from the printed dimension, e.g., the 9 mm samples became ~8.3 mm.

### 2.5. Fatigue Testing

Fatigue testing was conducted on all specimens according to ASTM E466 and occurred within roughly one month of the build performed. Prior to testing, the specimens were maintained within a desiccator at room temperature and pressure. The fatigue tests were performed using a commercial hydraulic universal testing system (Instron: Model 8520; Norwood, MA, USA) under constant amplitude load control with a stress ratio (R) of 0.1 and frequency of 20 Hz. The total number of cycles to failure was recorded for stress levels that were chosen to encompass a large range of the high cycle fatigue (HCF) regime below runout (10^7^ cycles). Due to the limited number of specimens in the as-built condition, only two stress levels were used to ensure that enough specimens were available to capture the statistical scatter in the fatigue life response for each specimen size and location.

For testing the fatigue specimens in the as-built condition maximum stress levels of 250 and 550 MPa were selected. These two levels are well within the HCF regime and allowed for fitting of the Basquin power law relationship. Similarly, for testing the machined fatigue specimens of the full factorial DOE, maximum stress levels 400, 600, 800, and 1000 MPa were selected. Stress–life diagrams were constructed with the fatigue responses and the Basquin model (Equation (1)) was fit to the data according to

(1) $$S=AN_{f}^{B}$$

where *S* is the measure of stress, *N_f_* is the number of cycles to failure, and *A* and *B* are the stress–life coefficient and exponent, respectively. The stress life distributions were evaluated with respect to the factors and analyzed statistically through multiple linear regression. The statistical analysis was performed using Minitab 14 (State College, PA, USA).

For comparison of results with the literature, the maximum stress was converted to an effective stress, as described by Li et al. [4], to correct data from studies that did not test at a stress ratio of 0.1. Converting to the effective stress with Equation (2) has been shown to be acceptable for −0.5 < R < 0.5 [4].

(2) $$S_{eff}=S_{max}{\left(\frac{1-R}{2}\right)}^{0.28}$$

### 2.6. Defect Analysis

Prior to fatigue testing, the porosity and its distribution within selected fatigue samples were evaluated using X-ray Computed Microtomography (XCT). The evaluations were performed using a commercial system (X5000, North Star Imaging, Rogers, MN, USA). The scan parameters used for all specimens are listed in Table 2. Projections were first reconstructed in a commercial software (efX CT 2.6, North Star Imaging, Rogers, MN, USA) that is provided by the manufacturer of the scanner. This software embedded the geometric data from the geometry tool that was scanned after the specimen. Once reconstructed, the volume is then exported as a slice stack along the build height direction. The image stack was then imported to VGSTUDIO MAX (Volume Graphics Inc., Charlotte, NC, USA) for evaluation of the porosity.

Based on results of the fatigue study with the as-built specimens, it was identified that an increased sample size was needed. Therefore, XCT analysis was conducted on >50% of the machined specimens and all combinations of the factor levels. To accommodate the increase in specimens that required scanning, multiple samples were scanned simultaneously to reduce the cumulative scan time. Placing an array of multiple samples in the scan region of interest led to changes of the scan parameters from those listed in Table 2. Firstly, the X-ray source voltage was increased to penetrate the extra material from having multiple specimens in line with the source and detector. Secondly, the geometric zoom was reduced to fit all the extra specimens into the detector window of view. Subsequently, these scans for the machined specimens have a spatial resolution of 16.49 µm, in comparison to 8.16 µm for the as-built specimens. Finally, placing more specimens in a single scan enabled longer scans with higher number of projections, while still maintaining a significant reduction in the time required per specimen. Using more projections improved the angular resolution of the scan planes and the grayscale accuracy for voxels further from the central axis of the part stage.

### 2.7. Fractography

A fractographic analysis of the fatigue specimens was performed post-failure using a combination of optical and scanning electron microscopy. Optical microscopy (OM) was performed on an Olympus Model SZX16 (Olympus, Center Valley, PA, USA) stereomicroscope for origin determination and qualitative mapping of the fracture surface. Scanning electron microscopy (SEM) was performed on a Philips Model XL-30 (Eindhoven, The Netherlands) using the secondary electron detector to resolve additional details of the fracture surfaces and perform quantitative measurements of the important features.

## 3. Results

### 3.1. Fatigue Life Responses

**Figure 3:**
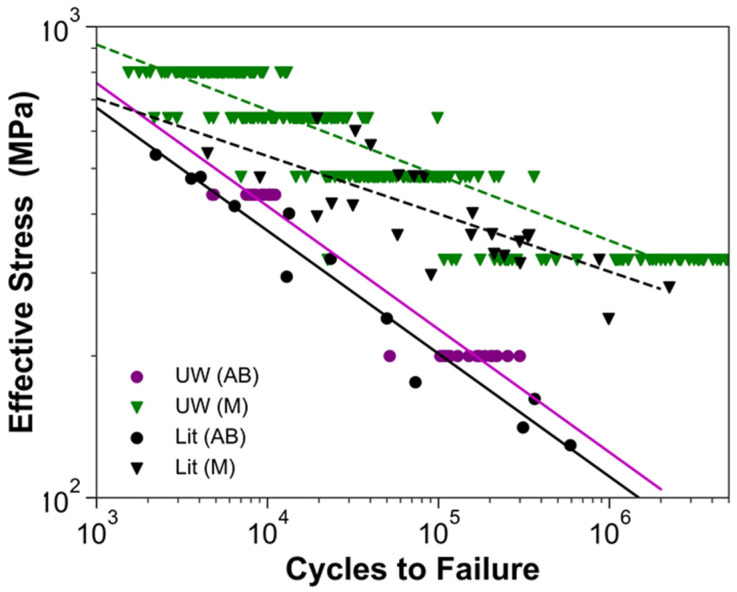
Stress–life (S-N) diagram for results of the present study with relevant EBM data from the literature for vertically oriented specimens [1].

Results from the experimental evaluation of the fatigue life are presented in Figure 3 along with data reported in the literature for Ti6Al4V produced by EBM AM [1]. As evident in this figure, there is a relatively large degree of scatter in the reported data. Nevertheless, results for the present study fall within the cluster of reported data for the as-built condition (AB), and for the machined (M) condition.

**Figure 4:**
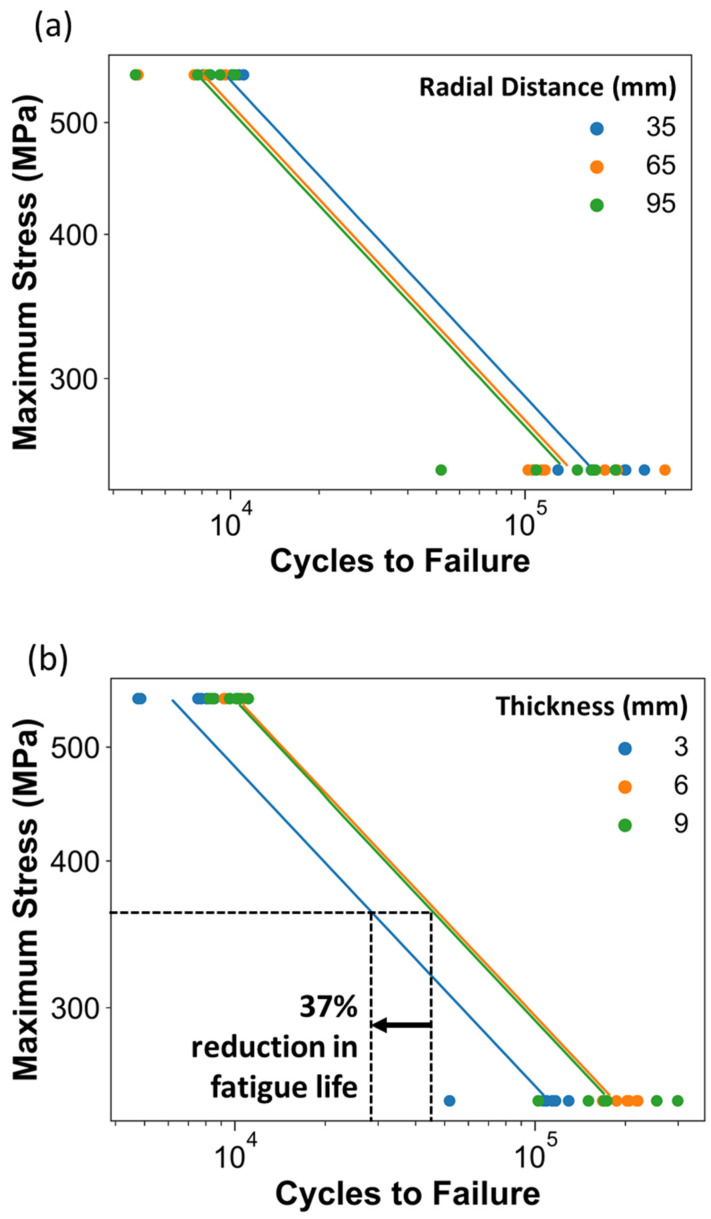
Stress–life diagrams of data from the DOE with metal in the as-built condition and with Basquin model fits. Shown are the effects of (**a**) radial distance, and (**b**) thickness factors.

Further scrutiny of the stress–life data according to the factor levels enables an assessment of the main effects. The fatigue data are presented with respect to the three levels of radial distance and thickness in Figure 4. Apparent from the distribution, there is a reduction in the fatigue life of the metal with an increasing radial distance from the center of the build plate (Figure 4a) and a substantial reduction (nearly 40%) in the fatigue life with a reduction in thickness from 9 to 3 mm.

**Figure 5:**
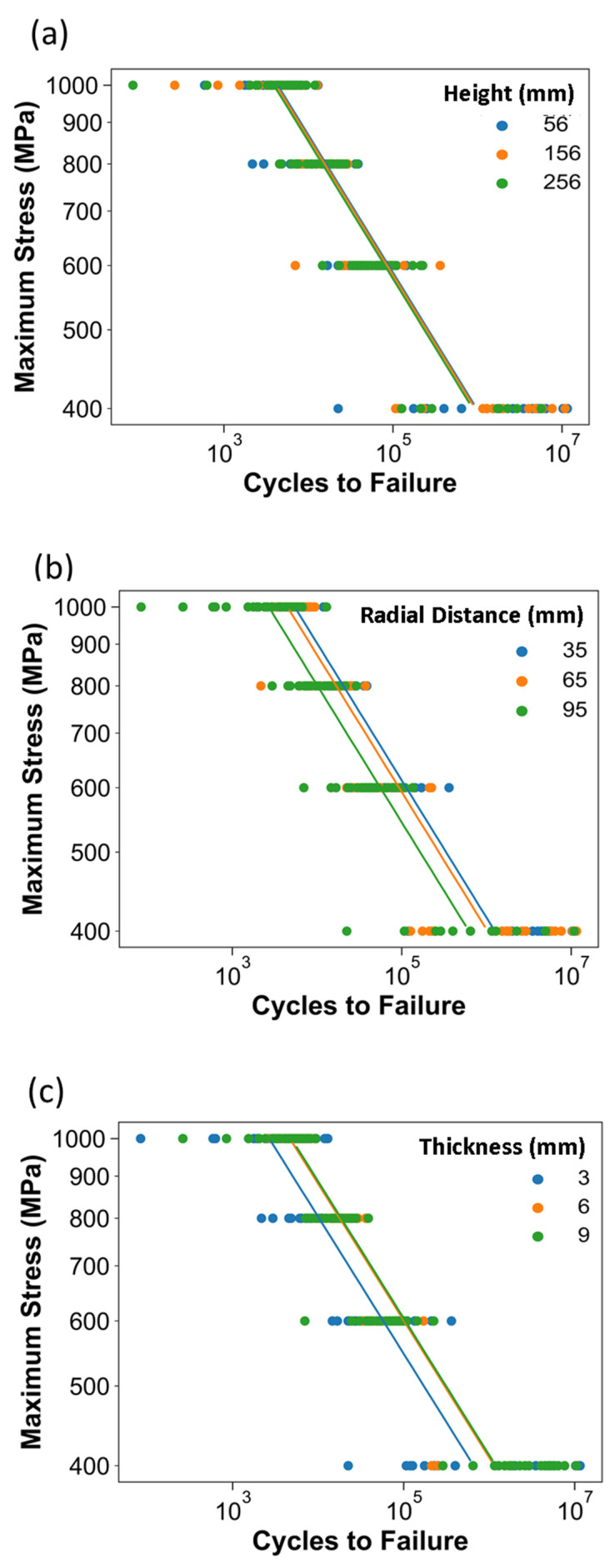
Stress–life diagrams for data (machined condition) from the DOE with Basquin model fits. Shown are the influence of (**a**) height, (**b**) radial distance, and (**c**) thickness factor levels.

The effects from the three primary factors on the fatigue life behavior of the metal in the machined condition are shown in Figure 5. Regarding the influence of height in Figure 5a, there is no significant difference between the fatigue life responses based on this factor at any of the stress levels. For the radial distance in Figure 5b, there is a substantial decrease in the fatigue life with an increase in the radial distance. Moreover, regarding the thickness in Figure 5c, the effects are akin to the as-built condition, i.e., the specimens with low thickness (3 mm) had a substantially lower fatigue life for all three stress levels.

### 3.2. Porosity

**Figure 6:**
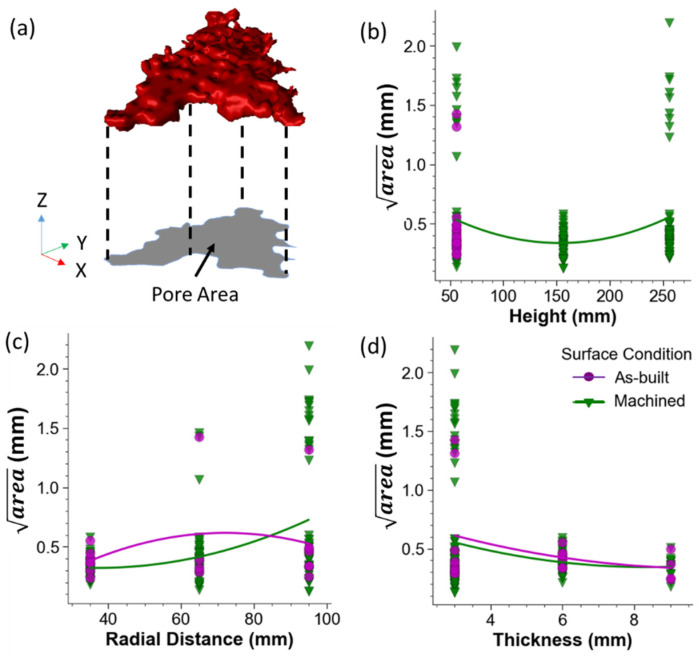
Pore size distribution in the metal of the DOEs. (**a**) The area of the pore (*A*) is defined as the area projected onto the plane perpendicular to the locating direction, which is the build plane (X–Y) for vertically-oriented specimens. (**b**–**d**) The maximum $\sqrt{area}$ of all pores in the gauge section of the specimens scanned with XCT as a function of the height, radial distance and thickness, respectively.

The severity of the pores can be defined in several ways including pore volume and the effective diameter. The largest pores in PBF are typically lack of fusion (LOF) voids that exhibit their largest dimension in-plane and exhibit high aspect ratios. As such, a description of the volume or diameter may underestimate the stress concentration. Spurious “rogue” defects that span many build layers also limit the applicability of these definitions. Originally proposed by Murakami [21] and applied to small defects in metallic materials [15], the root area of the pore is now among the prevalent methods for characterizing size and assessing their potential severity in AM [16,17,20]. An illustration concerning how the pore area is extracted is shown in Figure 6a.

The largest pore, characterized by the $\sqrt{area}$, for each scanned specimen are plotted in terms of the DOE factors in Figure 6. The importance of height on the pore size distribution of the metal in the as-built and machined conditions are presented in Figure 6b. As evident from this figure, there is an inverse quadratic relationship between the build height and the $\sqrt{area}$. Some samples exhibited exceptionally large internal rogue defects, which are not indicative of LOF-type voids. Interestingly, at the middle level height (156 mm) no samples scanned with XCT contained these large rogue defects.

The pore size distribution is shown in terms of the radial distance in Figure 6c. In general, for both the as-built and machined conditions the smallest pores are located at the center of the build space (i.e., at the smallest radial distance). The slight discrepancy in trends for the as-built and machined groups could be attributed to the smaller sample size for the as-built condition as only a single specimen in that condition contained a rogue defect, which was at the largest radial distance. For the machined condition, 44% of all machined samples with a 3-mm diameter and built with the largest radial distance had rogue defects.

**Figure 7:**
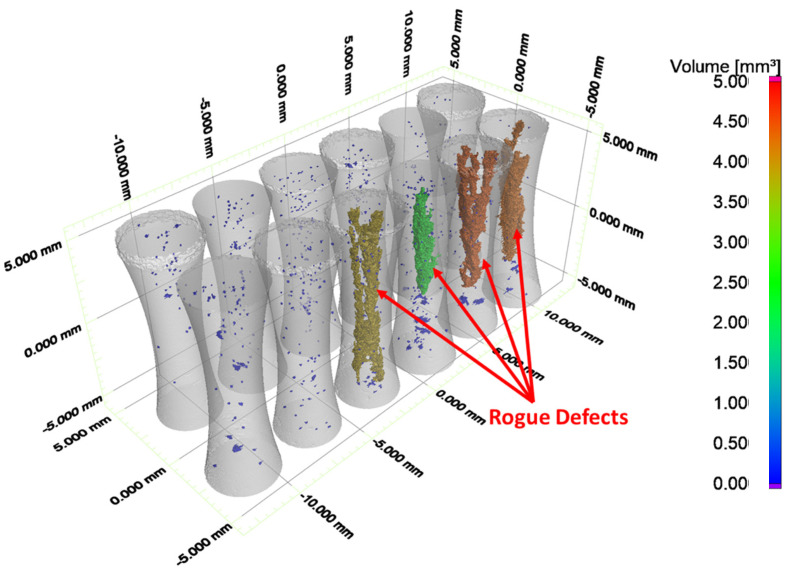
XCT models of machined samples from low-level diameter (3 mm) and highlight of pores with diameter ≥ 0.1 mm. Example “rogue” defects are highlighted. Diameter is defined by the minimum encompassing sphere, which is the longest linear dimension of the pore.

The dependence of the pore size on the part diameter is presented in Figure 6d, which shows that the largest pores were identified in specimens with the smallest thickness (3 mm). These specimens also exhibited a bimodal pore size distribution as the subset possessed very large pores (rogue defect) that extended over several layers of a build (Figure 7). If the rogue defects were excluded from the data set, the median measure of the largest pores for the parts with a 3-mm thickness would be lower than that for the specimens with a 6-mm thickness.

**Figure 8:**
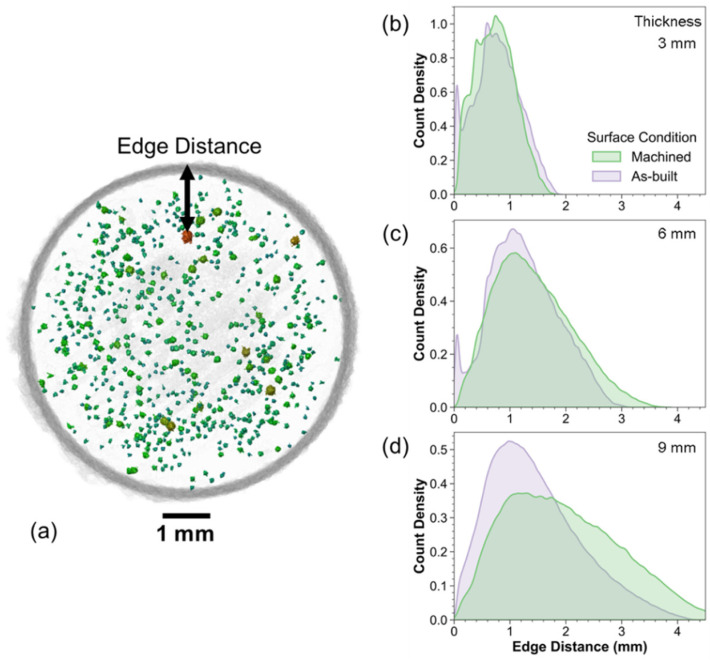
Pore distribution as a function of edge distance, which is defined as the distance of the surface of a pore to the free surface of the specimen as represented in the top view projection in (**a**). Count density plots for the three thickness levels (3, 6, and 9 mm) are shown in (**b**–**d**), respectively. The highest density of porosity is located at a ring around 0.7–1.1 mm from the surface.

The pore location relative to a free surface is also important due to the stress-state and the time necessary for inherent damage to transition into a crack. Hence, the edge distance was defined and quantified by the shortest radial distance from the pore surface to the part surface in the plane perpendicular to the loading direction (Figure 8a). Histograms were constructed to assess the pore distribution as a function of the edge distance, for the three specimen sizes in Figure 8b,c. Regardless of thickness, the overall maximum pore concentration in the as-built condition was evident at an edge distance of approximately 0.7–1.1 mm from the free surface. A local maximum in the pore density was noted near the surface (<0.2 mm from the surface) in the 3- and 6-mm-thick specimens of the as-built condition. Machining removed the near-surface pores, which is evident from the absence of this peak in the histograms. Interestingly, a local maximum was not evident in the histogram for the 9-mm-thick specimens (Figure 8d).

**Figure 9:**
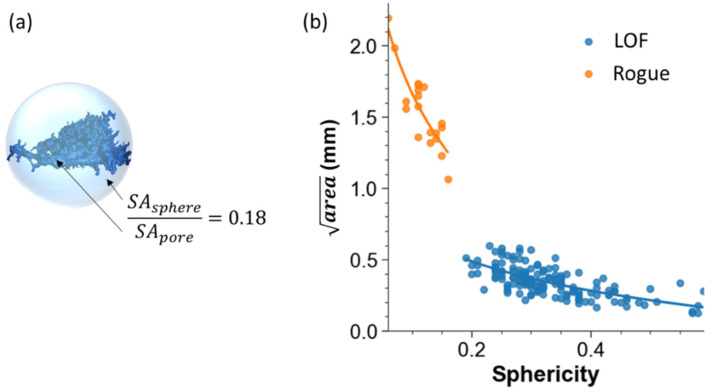
Sphericity of internal pores and voids. (**a**) Example of a LOF defect and the calculated sphericity according to Equation (3). (**b**) Pore size distribution in terms of the $\sqrt{area}$ as a function of sphericity. Note the separation of the rogue and LOF defects.

The final metric for describing the pore geometry is morphology, which can be described in terms of its sphericity, aspect ratio, and radius of curvature. The sphericity defines the ratio of the surface area of the pore to the surface area of a sphere with an equivalent volume, as shown in Figure 9a. Sphericity is defined as

(3) $$Sphericity=\frac{SA_{sphere}}{SA_{pore}}=\frac{\pi^{\frac{1}{3}}{\left(6V_{pore}\right)}^{\frac{2}{3}}\;}{SA_{pore}}$$

where *SA* is the surface area and *V* is the volume.

As evident in Figure 9b, rogue defects and LOF voids exhibit an exponential relationship with sphericity, which is consistent with Electron Beam PBF of Ti6Al4V [18]. Specifically, there is a decrease in the sphericity of the pores/voids with increasing size, which is of substantial concern.

### 3.3. Fractography

**Figure 10:**
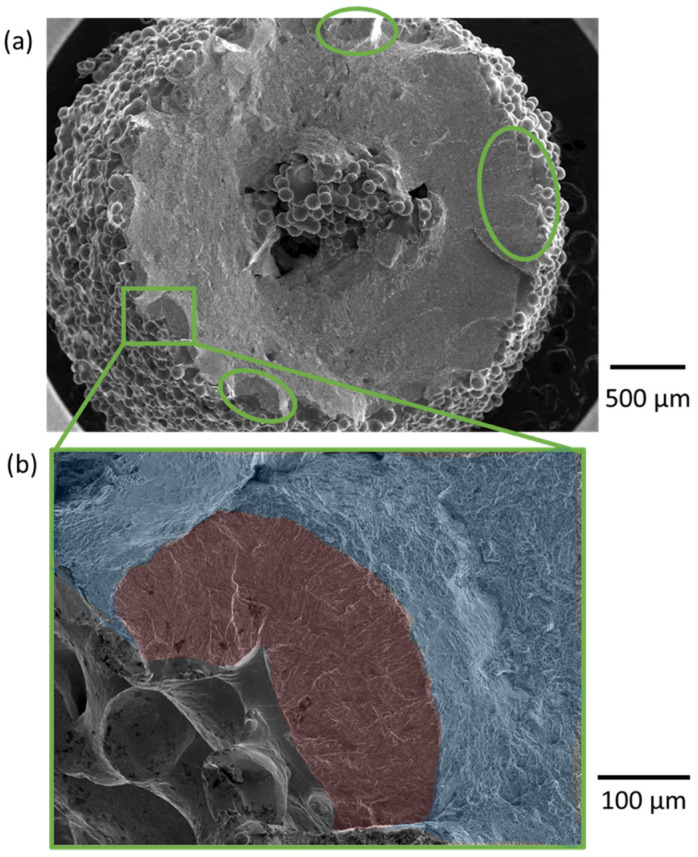
Fractographs of a representative as-built specimen with 3-mm thickness (low-level). (**a**) Low magnification SEM view. Despite the large pore at the center of the specimen, multiple origins were identified along the surface. (**b**) Higher resolution micrograph indicating fatigue crack initiation from the surface. Red: Stage II fatigue crack propagation zone. Blue: Stage III fast fracture.

An evaluation of the fracture surfaces of exemplary specimens was conducted to determine the origin of fatigue failure. A micrograph of the fracture surface for an as-built specimen with rogue defect is shown in Figure 10a. Despite the presence of a rogue defect that occupied up to 10% of the fracture surface area, the failure origins of the as-built specimens with these defects were found to be at the exterior surface.

For the specimens evaluated in the as-built condition, the origins of fatigue failure were surface flaws, as shown in Figure 10b. Some stable crack growth is visible (red region) and distinguished by the transition between a diffuse fracture mirror and the transition from radial lines to hackle, extending perpendicular to the loading axis. Once a sufficient number of cracks were initiated from the surface and progressed to a critical size, fast fracture (blue region) ensued, characterized by the stereotypical dimpling of ductile titanium under monotonic loading.

**Figure 11:**
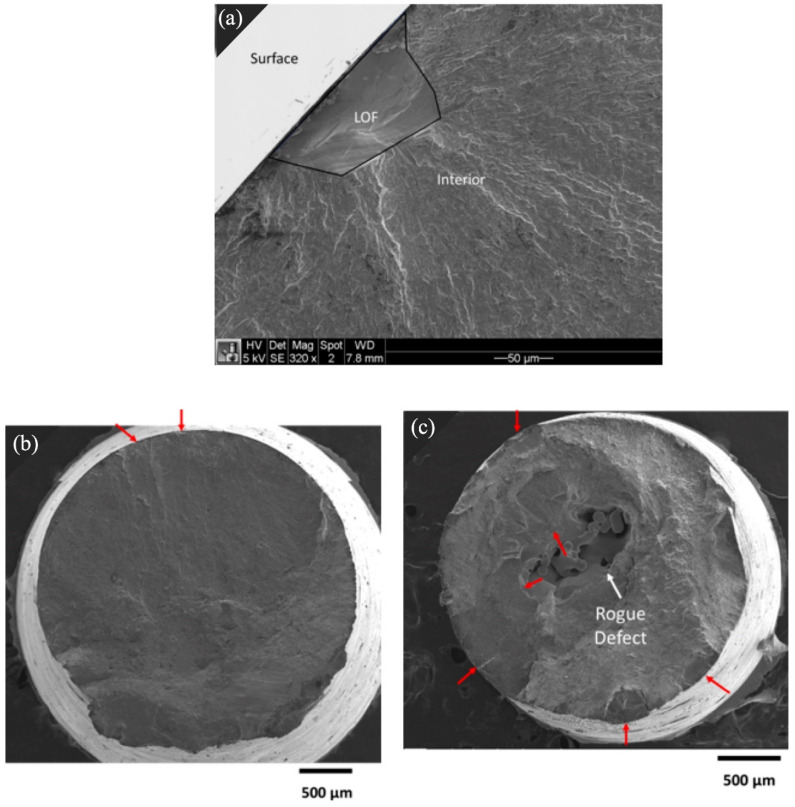
SEM fractographs of (**a**) LOF defect that was exposed to the surface after machining, (**b**) a low porosity sample exhibiting two dominant origins at the surface, both exposed LOF defects, and (**c**) multiple surface origins in a specimen with large rogue defect at the center. Both (**b**,**c**) are from machined 3-mm thickness specimens.

The removal of the as-built surface texture by machining did not prevent the surface origins of fatigue failure as the material removal exposed sub-surface LOF defects, as shown in Figure 11a. Representative origins of fatigue failure evident in the fracture surface of the machined fatigue specimen are shown in Figure 11b,c. In Figure 11b, a low porosity sample is shown that exhibits at least two dominant origins at the surface, as highlighted. A second example in Figure 11c exhibits multiple originals of fatigue, including at the surface, despite the large interior rogue defect. The surface origins appear to involve voids that were exposed by material removal.

## 4. Discussion

As evident from the stress–life diagrams in Figure 3, the fatigue data from the present investigation compared favorably with that reported in the literature for the EBM of Ti6Al4V. There was substantial scatter in the experimental data, which was at least partly attributed to the range in the design parameters. Much of the scatter in the reported data is associated with differences in the surface condition and specimen orientation; post-build machining and the horizontal orientation of specimens (aligned to the build plane) generally exhibited a superior fatigue life. If the comparison with reported fatigue data is restricted to the vertical orientation, the Basquin models developed from the experimental data for the as-built and machined conditions are in close agreement with the literature. As few published studies report the beam parameters, machine type, or powder condition [1,4,22], there are limitations to these comparisons. Nevertheless, despite the difficulty in accounting for all the potential sources of difference in the fatigue life distributions, the consistency in the experimental and reported data helps build confidence.

**Figure 12:**
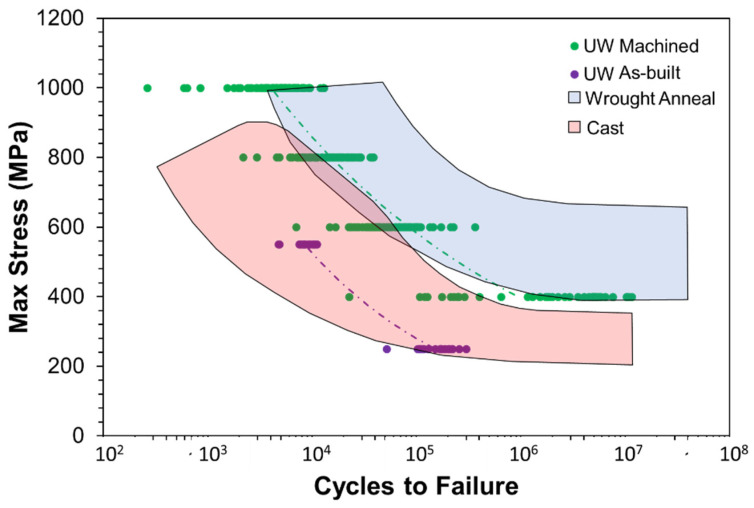
Stress–life diagram comparing the data generated during the fatigue DOE study in both the as-built and machined states against fatigue life range for two traditional Ti6Al4V materials. Fatigue life distributions are extracted from the ASM Handbook [23].

Casting and wrought forming are the primary manufacturing processes for Ti6Al4V components in aerospace. One goal for metal AM is to reach an equivalent or superior performance to that achieved by wrought forming. A comparison of the fatigue data from the DOE and that from published sources for cast and wrought conditions is shown in Figure 12. Results for the as-built condition fall within the region of fatigue life data reported for casting [23]. While results for the machined condition are superior to the cast condition, they are not consistent with that for wrought form metal. Furthermore, a portion of the machined data falls within the region of cast metal, which corresponds to the EBM specimens with rogue defects. Assuming process control could eliminate rogue defects, then the fatigue life responses for the machined condition are comparable to that of Ti6Al4V manufactured by wrought forming and in the annealed condition. Further improvements could be garnered by employing a post-process treatment such as solution heat treating and aging (STA) or hot isostatic pressing (HIP) [1,24].

Build design parameters were found to have a significant effect on the fatigue properties of the printed specimens in both the as-built and machined condition. Of the factors evaluated, the most significant effect was attributed to the part’s thickness. There was nearly a 40% reduction in the as-built fatigue life of the specimens with a 3-mm thickness with respect to those with a 6- and 9-mm thickness. Similarly, for the machined condition, the fatigue life decreased with the decreasing thickness, with an average reduction of approximately 45% across the three stress levels. Some specimens exhibited subsurface LOF voids and surface defects that worked synergistically as the origin of failure, while others had only LOF or surface defects. Le et al. [12] investigated the effects of part size (thickness) on the fatigue life of Ti6Al4V produced by PBF and reported that machined specimens can have many different complementary failure origins that expand the scatter in the fatigue responses.

Specimens with the smallest diameter (3 mm) had a lower fatigue life due to two primary factors. First, the 3-mm diameter was the only size that exhibited rogue defects. These exceptionally large defects were located at the center of some of the 3-mm specimens and resulted in larger effective stress in the net cross-section. Secondly, the stress intensity posed by a crack in the specimens with small cross-section is amplified due to the boundary effects, which increase the cyclic growth rate and result in a lower propagation life [25,26]. Another factor to consider is the increased surface roughness of printed parts with thinner nominal dimensions [19]; the increased roughness of thin specimens was sufficient to reduce the fatigue life [8,19].

The fatigue life decreased with the increasing radial distance for both the as-built and machined conditions. Unlike the underlying effect from the part thickness, the effects of the radial distance were not related to the rogue defects. There are several potential explanations for this trend. First, the electron beam exits from the column hundreds of millimeters above the build layer. The beam’s angle of incidence with the powder bed increases as the distance from the center of the build plate increases, i.e., increasing radial distance. The larger incident angle [27] will decrease the energy absorbed by the metal powder, which will reduce the penetration of the melt pool [28] and facilitate LOF defects [10]. Another possibility is the greater potential for irregularities in the powder spreading at the outermost radial distance. If underdosing occurs, the powder that spreads to the opposite side of the fetching could be sparse or incomplete. Low density powder layers result in defects since an inadequate amount of material is available to be melted [29]. However, insufficient powder spread is unlikely in this investigation since it occurred over three separate builds. Each build requires a rake position calibration during which the operator ensures the powder fully covers the build plate. Furthermore, the ARCAM A2X has a feedback loop system to automatically adjust the rake position to compensate for low powder dosing.

The rogue defects played a detrimental role to the fatigue life distributions and there are three ways in which they caused a reduction in the fatigue life of the metal in the as-built condition. First, they occupied a substantial portion (>10%) of the cross-section and thereby amplified the axial stress, which was not accounted for in the estimated cyclic stress. Secondly, the geometry of these defects caused an internal stress concentration that further increased the localized stress. And thirdly, after fatigue crack initiation from the various surface origins, the growth rate was increased due to the higher stress and stress intensity [30,31]. This combination of factors was responsible for the reduction in the fatigue life of the specimens with the lowest thickness (3 mm) in relation to the other two levels and would apply to the structural components with small cross sections. However, how should their contribution to the fatigue response be accounted for?

The severity of the pores/voids is a complicated metric to define. Several studies that have pursued an evaluation of defect severity for AM metals have applied both stress intensity and stress concentration definitions. The most prevalent of the treatments applied a modified linear elastic fracture mechanics (LEFM) approach to inclusions and pores in metals [15]. Murakami and Endo [21] proposed a stress intensity description to micro-scale defects according to

(4) $$K=FS\sqrt{\pi \sqrt{area}}$$

where “*area*” is the projected area of the defect onto the plane perpendicular to the direction of loading, as shown in Figure 6. According to this quantitative description, it would be expected that the trend in the fatigue life would be inversely related to the defect size based on the increase in the apparent stress intensity. Indeed, the as-built and machined specimens with a 3-mm thickness that contained rogue defects demonstrated the worst fatigue performance. Yet, they did not serve as the origin of failure as fractographic markings showed that the origins were located at the surface (Figure 11). Hence, these internal defects were detrimental, but it was not the apparent stress intensity of these defects that caused a reduction in the fatigue life.

Although several studies have applied the $\sqrt{area}\;$ treatment of pores to assess the mechanical behavior of AM metal [12,16,17], there are limitations to this approach. Murakami et al. investigated the effects of small defects on the fatigue performance of metallic materials extensively, including the application of the $\sqrt{area}\;$ definition [32,33,34]. They have acknowledged the limitations of the $\sqrt{area}$ parameter [7], especially for very irregular pores, and worked to expand upon the original description [21]. Indeed, the defects produced by EBM can be complex and accounting for the projected area alone on the plane of maximum normal stress is potentially an oversimplification. The Murakami equation (Equation (4)) does not capture the effects of the specific detrimental attributes of the pores, such as location, geometry, and clusters [7,11,13,16]. Further improvements are necessary to obtain a model that can accurately predict the fatigue life from the defect distributions.

Finite element analysis of micro-scale defects has shown that the stress concentration of internal voids will increase substantially as they approach the surface of the component [11,16]. These “near-surface” defects, can pose stress concentrations that are multiple times greater than internal defects located far from the free-surface boundaries. There is a sharp increase in the stress concentration when the ratio of the distance between the defect and part surface to the pore diameter decreases below 1 [16]. Furthermore, the magnitude of the stress concentration grows asymptotically as the distance ratio approaches 0. Thus, post-process machining that is applied to improve surface quality can inadvertently transition internal pores to “near-surface” defects and have detrimental consequences.

To determine the relative contributions of pore characteristics to the fatigue responses, linear regression was applied to the XCT porosity and fatigue data. A power law relationship was found between the defects and cycles to failure in the present data that corroborates findings from other researchers [16]. A log transformation of the power law equation results in a linear relationship and allows for the application of linear regression. A semi-empirical equation for the fatigue life in terms of the Basquin model and three pore characteristics that reflect the size, location, and morphology is given by

(5) $$\begin{array}{ll} log_{10}\left(Nf\right)= & 21.89-6.20(log_{10}\left(S_{max}\right))-0.29(log_{10}\left(\sqrt{area}\right))\\ & +\;0.40\left(log_{10}\left(Sphericity\right)\right)+0.11\left(log_{10}\left(Edge\;Distance\right)\right) \end{array}$$

**Figure 13:**
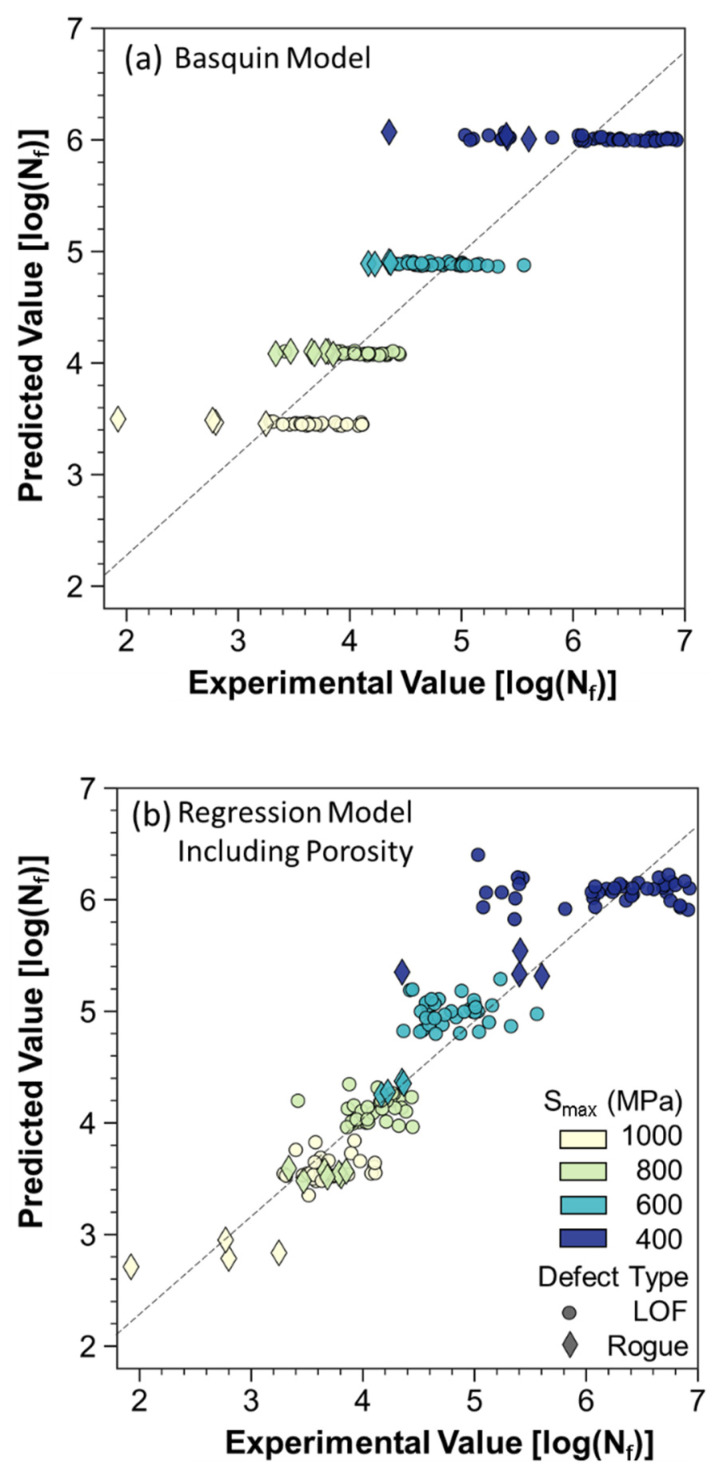
Comparison of predicted values against experimental values for (**a**) the Basquin model and (**b**) the linear regression model that accounts for porosity characteristics (Equation (5)).

As evident from the fit coefficients, the stress level (*S_max_*) accounts for the major portion of the fatigue life response in the model. The addition of the pore characteristics enhances the model to encompass portions of the data that are influenced by these parametric effects. A comparison of the experimental fatigue life responses with the traditional Basquin model that does not account for porosity effects is shown in Figure 13a. Results for the application of the fatigue life relationship in Equation (5) are shown in Figure 13b. As evident in this figure, the specimens with rogue defects are more consistent with the regression fit and exhibit a similar effect to having a specimen at a higher stress level. In general, these extreme defects posed a greater maximum cyclic stress and stress amplitude that reduced the fatigue life. Based on the model, the effective increase in stress caused by these defects is approximately 200 MPa. Even for the specimens with only LOF defects, the data are spread along the regression line, implying that the stress concentration posed by the largest pores in these specimens causes an amplification of the applied stress level.

Utilizing XCT data to model or manage the influence of porosity/voids on the fatigue life response appears promising. However, there are clear limitations to this approach. One concern is that surface defects are generally not captured in these evaluations of porosity since post-process rendering only characterizes defects that are enclosed by material. In addition, all specimens had contributing surface origins, which are not included in the regression model and possibly contribute to the variability in the data. A notch root radius calculation could be used to estimate the maximum stress concentration of the surface or identify voids with sharp tips that should be treated as a stress intensity. Yet, that requires an inspection technique with resolution capable of capturing the notch root radius. Another concern is that the quantitative descriptions of porosity are generally limited by the way the metrics can be assessed. For instance, the proximity of pores could contribute to the fatigue life [7,16]. The current algorithm for quantifying the proximity of the pores in the 3D space is based on the distance of minimal encompassing spheres. The limitation is that the distance is the nearest vectorial distance and not defined with respect to the component or build coordinate directions. As such, this proximity metric has limitations in assessing the defect severity and was neglected in the current model. A description for the proximity of pores within the same plane (i.e., plane of maximum normal stress), or within adjacent build layers are more relevant [7]. Therefore, complementary work focused on defect geometry, defect interactions, and their contribution to the fatigue performance of EBM and other AM metals could be valuable.

## 5. Conclusions

Despite substantial focus on the contributions of process parameters to the fatigue life and part reliability in metal powder bed fusion processes, limited effort has been placed on understanding the influence from design parameters. This investigation was focused on determining the effects of key build design parameters, including the part thickness and part location in the build space, on the fatigue performance of Grade 5 Ti6Al4V produced by EBM. The conclusions are:

- Part diameter had significant effects on the fatigue life of the metal in both the as-built and machined conditions. The fatigue strength decreased with decreasing specimen diameter, which resulted from the increase in defect size with respect to the part cross-section. The smallest diameter specimens (3 mm) had a greater number of large internal “rogue” defects, which increased the effective stress and served as origins for fatigue crack initiation.
- The radial distance of parts within the build envelope had significant effects on the resistance to fatigue failure. The fatigue strength decreased with the increasing radial distance from the center of the build plate.
- In the as-built condition, the surface texture consistently served as the origin of failure. Stress concentrations were posed by the surface texture that served as sites for fatigue crack initiation and caused substantial reductions in the fatigue life. Machining of the surface improved the fatigue resistance and resulted in a part performance which was more comparable to wrought annealed Ti6Al4V.
- A post-process surface treatment is essential for metal components produced by EBM that are intended for stress-critical applications. However, it is critical to understand the location of the internal pores/voids to optimize the post-process machining. The depth of machining must remove the defects inherent of the surface but avoid relieving the near-surface defects that are concentrated beneath the free surface.
- Further analysis of the defect distributions is necessary to develop predictive models that can account for their contribution to the fatigue properties. The important parameters include the pore/void size, their location relative to the surface of the part, the in-plane morphology and their proximity to each other. In addition to internal defects, it is essential to account for pores that are relieved by net-shape machining, which are not adequately identified by XCT.

## Figures and Tables

**Table 1:** DOE factor levels of the three build design parameters. Red numbers indicate factor levels that only exist in the machined DOE.

Level	Height (mm)	Radial Distance (mm)	Thickness (mm)
Low	56	35	3
Mid	156	65	6
High	256	95	9

**Table 2:** Parameters used for scanning as-built fatigue samples.

Scan Parameter	As-Built	Machined
Voltage	160 kV	220 kV
Current	60 µA	75 µA
Geometric zoom	23.33X	11.82X
Projections	1200	5400
Scan mode	Step	Step
Frame average	2 frames/projection	2 frames/projection
Frames per second	1 fps	2 fps
Gain maps	7	7
Resolution	8.162 µm	16.49 µm
Filter	0.005 in. brass	0.005 in brass

## Data Availability

Data for this study is proprietary.
